# Duplication-based genetic dissection of the Down syndrome critical region reveals its complex functional organization

**DOI:** 10.1093/g3journal/jkag173

**Published:** 2026-07-01

**Authors:** Xiaoling Jiang, Chunhong Liu, Zhuo Xing, Yichen Li, Avrium Douglas, Lingqiu Gao, Annie Pao, Jill Silverman, Y Eugene Yu

**Affiliations:** The Children's Guild Foundation Down Syndrome Research Program, Department of Cancer Genetics and Genomics, Roswell Park Comprehensive Cancer Center, Buffalo, NY 14263, United States; The Children's Guild Foundation Down Syndrome Research Program, Department of Cancer Genetics and Genomics, Roswell Park Comprehensive Cancer Center, Buffalo, NY 14263, United States; The Children's Guild Foundation Down Syndrome Research Program, Department of Cancer Genetics and Genomics, Roswell Park Comprehensive Cancer Center, Buffalo, NY 14263, United States; Genomic Medicine Institute, Lerner Research Institute, Cleveland Clinic, Cleveland, OH 44195, United States; The Children's Guild Foundation Down Syndrome Research Program, Department of Cancer Genetics and Genomics, Roswell Park Comprehensive Cancer Center, Buffalo, NY 14263, United States; The Children's Guild Foundation Down Syndrome Research Program, Department of Cancer Genetics and Genomics, Roswell Park Comprehensive Cancer Center, Buffalo, NY 14263, United States; The Children's Guild Foundation Down Syndrome Research Program, Department of Cancer Genetics and Genomics, Roswell Park Comprehensive Cancer Center, Buffalo, NY 14263, United States; MIND Institute, University of California Davis School of Medicine, Sacramento, CA 95817, United States; Department of Psychiatry and Behavioral Sciences, University of California Davis School of Medicine, Sacramento, CA 95817, United States; The Children's Guild Foundation Down Syndrome Research Program, Department of Cancer Genetics and Genomics, Roswell Park Comprehensive Cancer Center, Buffalo, NY 14263, United States; Genetics, Genomics and Bioinformatics Program, State University of NewYork at Buffalo, Buffalo, NY 14263, United States

**Keywords:** Down syndrome critical region, chromosome engineering, mouse models, genetic dissection, cognitive deficits

## Abstract

Down syndrome (DS), associated with trisomy 21, is the most common genetic cause of developmental delay and intellectual disability, yet the specific dosage-sensitive genes and the associated genetic mechanisms underlying these phenotypes remain incompletely defined. Here, we applied an additive genetic strategy to dissect the Down syndrome critical region (DSCR) by generating 2 complementary mouse models using Cre/loxP-mediated chromosome engineering that together span the entire DSCR on mouse chromosome 16: Dp(16)5Yey, duplicating the *Setd4*–*Kcnj6* interval, and Dp(16)6Yey, duplicating the *Kcnj15*–*Mx2* interval. In addition, we engineered a third duplication model, Dp(16)7Yey, carrying a selective duplication of the *Dyrk1a*–*Kcnj6* interval containing only these 2 genes. Building upon our previously reported results, cognitive behavioral analyses of these 3 models reveal a complex functional genetic architecture of the DSCR, including dosage-sensitive genetic elements, interactions among these elements, and their contributions to DS-associated cognitive deficits. Together, these findings highlight the complexity of dosage-dependent genetic interactions, which provide important insights into DSCR functional organization and have major implications for the development of effective therapeutic strategies for DS-associated cognitive deficits. In addition, these duplication mouse models represent valuable resources for further genetic dissection of DS phenotypes beyond cognition.

## Introduction

Down syndrome (DS), associated with trisomy 21, is among the most common chromosomal disorders, occurring in ∼1 in 691 live births (Parker et al. 2010) and affecting ∼1 in 1,499 individuals in the United States (de Graaf et al. 2017). DS is the leading genetic cause of developmental delay and intellectual disability, yet the underlying dosage-sensitive genes and the associated genetic mechanisms remain incompletely defined (Antonarakis et al. 2020).

Following the identification of trisomy 21 (Lejeune et al. 1959a, 1959b), early efforts in Europe and the United States analyzed individuals with segmental trisomies to establish genotype–phenotype relationships (Korenberg 1990; Delabar et al. 1993; Sinet et al. 1993; Korbel et al. 2009; Lyle et al. 2009). However, the small number of informative cases has limited the ability of human genetics alone to identify causative genes for most DS-associated phenotypes.

Mouse models provide a powerful complementary approach because regions orthologous to human chromosome 21 (Hsa21) are conserved on mouse chromosomes 10, 16, and 17, and triplication of these regions recapitulates many DS-related phenotypes (Yu et al. 2010; Herault et al. 2017; Lana-Elola et al. 2021). Over the past 2 decades, a large collection of duplications (Dp) and deletions (Df) spanning Hsa21-orthologous intervals has been generated (Xing et al. 2016; Herault et al. 2017; Lana-Elola et al. 2021), enabling systematic genetic dissection.

Two complementary strategies can be applied to define dosage-sensitive genes. The subtractive strategy normalizes specific subregions within a large triplicated genomic region, and phenotypic rescue indicates that the deleted interval or gene contributes to the phenotype (Salehi et al. 2006; Duchon et al. 2011; Garcia-Cerro et al. 2014; Jiang et al. 2015; Marechal et al. 2015; Sawa et al. 2022). The additive strategy, in contrast, assesses the phenotypic consequences of triplicating defined genomic intervals (Pereira et al. 2009; Lana-Elola et al. 2016; Watson-Scales et al. 2018; Redhead et al. 2023; Sloan et al. 2023). Although individual genes can have direct effects, many DS phenotypes likely arise from interactions among triplicated genes, as supported by both predicted and experimentally demonstrated interactions (Ma'ayan et al. 2006; Siddiqui et al. 2008; Pereira et al. 2009; Sturgeon et al. 2012; Zhang et al. 2014; Jiang et al. 2015; Marechal et al. 2015; Duchon et al. 2021).

In our previous work, we applied a mouse-based subtractive strategy to analyze the Down syndrome critical region (DSCR). Here, we perform the complementary additive analysis by engineering and analyzing triplications of 3 defined genomic regions in mice to determine the phenotypic consequences of increased gene dosage within this critical portion of the DS genomic landscape.

## Materials and methods

### Generation of duplication mouse mutants

Cre/loxP-mediated chromosome engineering (Ramirez-Solis et al. 1995; Yu and Bradley 2001) was used to generate new chromosomal duplications. Specific MICER clones (Adams et al. 2004) were selected for use as the targeting vectors to deliver loxP to the 2 endpoints of each duplication in the genome of mouse ES cells, i.e. AB2.2 ES cells (Bradley et al. 1998). Prior to gene targeting, MICER clones with the mouse genomic DNA inserts were linearized with specific restriction enzymes. The linearized targeting vectors were electroporated into ES cells, which were then selected with G418 or puromycin. Double-targeted ES cell clones were identified by Southern blot analysis using PCR products as probes. ES cell culture and electroporation were carried out as described (Ramirez-Solis et al. 1993). To induce recombination between targeted loxP sites, a Cre-expression vector, pOG231 (O'Gorman et al. 1997), was electroporated into double-targeted cells. ES cell clones carrying individual duplications were identified by Southern blot analysis using ES cell DNA digested with specific restriction enzymes and hybridized with specific probes and confirmed by fluorescent in situ hybridization (FISH) analysis. The ES cell lines carrying the aforementioned individual duplications were used to generate germline chimeras by injecting them into blastocysts isolated from C57BL/6J mice, as described previously (Bradley 1987; Ramirez-Solis et al. 1993; Yu et al. 2006). Duplication mutant mice were identified by Southern blot analysis of mouse tail DNA.

### Animals

Mutant mice carrying Dp(16*Setd4*-*Kcnj6*)17Yey (Dp(16)5Yey), Dp(16*Kcnj15*-*Mx2*)18Yey (Dp(16)6Yey), or Dp(16*Dyrk1a*-*Kcnj6*)19Yey (Dp(16)7Yey) were first established in a (129Sv × C57BL/6J)F1 background and backcrossed to C57BL/6J mice for 5 generations. All mice were maintained in a temperature- and humidity-controlled animal facility with a 12-h light/dark cycle and had ad libitum access to food and water. All mice used in the behavioral experiments were 2 to 4 months old. Before behavioral experiments, each mouse was pre-handled for 2 min every day for 1 week. All experimental procedures were approved by the Institutional Animal Care and Use Committee of Roswell Park Comprehensive Cancer Center, and all animal experiments were conducted in accordance with the ARRIVE guidelines (Percie du Sert et al. 2020).

### Fluorescent in situ hybridization (FISH)

FISH analysis was performed on interphase nuclei of ES cells as described previously (Robertson 1987; Yu et al. 2006). To detect chromosomal duplications between *Setd4* and *Kcnj6*, between *Kcnj15* and *Mx2*, or between *Dyrk1a* and *Kcnj6* on Mmu16, individual BAC clones containing mouse genomic DNA mapped within these regions were selected, labeled with digoxigenin, and detected using anti-digoxigenin rhodamine antibodies. A BAC clone containing mouse genomic DNA mapped to Mmu16 was used to identify Mmu16, labeled with biotin, and detected with fluorescein isothiocyanate–avidin. Chromosomes were counterstained with 4′,6-diamidino-2-phenylindole (DAPI). FISH images were acquired using a Nikon Eclipse 80i fluorescence microscope (Nikon, Tokyo, Japan) equipped with a charge-coupled device (CCD) camera and a 100× oil-immersion objective. Images were acquired sequentially for each channel using exposure times of 100 ms for DAPI, 200 ms for fluorescein isothiocyanate (FITC), and 300 ms for cyanine 3 (Cy3). Identical exposure settings were used for all images within each channel.

### Real-time quantitative RT-PCR

Real-time quantitative polymerase chain reaction (RT-qPCR) was used to analyze expression of selected genes in mice carrying different genotypes. *Gapdh*, located on Mmu6, was used as the internal control for all mice examined. Total RNA was isolated from mouse brains using TRIzol Reagent (Life Technologies, Grand Island, NY, USA) and reverse-transcribed into cDNA using SuperScript III reverse transcriptase (Life Technologies). For quantitative RT-PCR analysis, cDNA samples from 3 mice per genotype were analyzed using gene-specific probes from the TaqMan Gene Expression Assays System (Life Technologies). PCR reactions were carried out on a StepOnePlus Real-Time PCR System (Life Technologies) under the following conditions: an initial activation and denaturation at 95 °C for 10 min, followed by 40 cycles of denaturation at 95 °C for 15 s and annealing/extension at 60 °C for 1 min.

### T-maze test

T-maze tests were performed on the basis of the protocol of Deacon and Rawlins (Deacon and Rawlins 2006) to examine hippocampal function. The T-shaped maze was made of opaque Plexiglas with a sliding door separating the start arm (50 cm × 10 cm × 20 cm) from the rest of the apparatus, which comprised a choice point at the intersection of the start arm with the left and right arms of the maze (37 cm × 10 cm × 20 cm for each arm). A divider panel (20 cm × 20 cm), which extended 10 cm into the start arm, was centered in the middle of the “T” between the left and right arms. Each mouse was placed into the start arm for 5 min of confined time, then the sliding door was opened and the mouse had free access to the rest of the maze for 10 min. Any entry into one of the lateral arms was recorded, and entry into an opposite arm was defined as an alternation. Only valid entries were counted (i.e. the whole body was inside the lateral arm, including its tail). The percentage of times the mouse alternated between the left and right arms over the possible alternations (total entry number minus one) was defined as the alternation rate.

### Contextual fear conditioning test

The contextual fear-conditioning test was performed as described previously (Jiang et al. 2015). Briefly, each mouse was given 2 min to explore the test chamber (baseline activity). The floor was a grid of stainless-steel rods connected to an electric shock generator. A video camera was mounted in the front of the chamber, and a ceiling light illuminated the interior through the transparent ceiling. Delivery of a foot shock (1 mA scrambled) for 2 s was controlled by Video Freeze Software (Med Associates, St. Albans, VT, USA). The mouse was allowed to stay in the chamber for an additional 30 s and then removed. Approximately 24 h later, each mouse was returned to the chamber and monitored for freezing behavior for 3 min, without any foot shock being delivered. Freezing behavior was recorded automatically by Video Freeze Software. Mean freezing activity during the contextual exposure was calculated as a measure of contextual learning.

### Statistical analysis

GraphPad Prism 10 (GraphPad Software, Boston, MA, USA) was used to perform statistical analyses. Data from the T-maze and contextual fear-conditioning tests were analyzed with the Student's *t*-test. In the behavioral experiments, *n* indicates the number of mice. All data reported in the text and figures are expressed as the mean ± standard error of the mean. Sample sizes are provided in the figure legends, with statistical significance reported in the text and indicated in the figures.

## Results and discussion

### Engineering and phenotypic analysis of mice carrying a triplication spanning the *Setd4–Kcnj6* genomic region

**Fig. 1.**
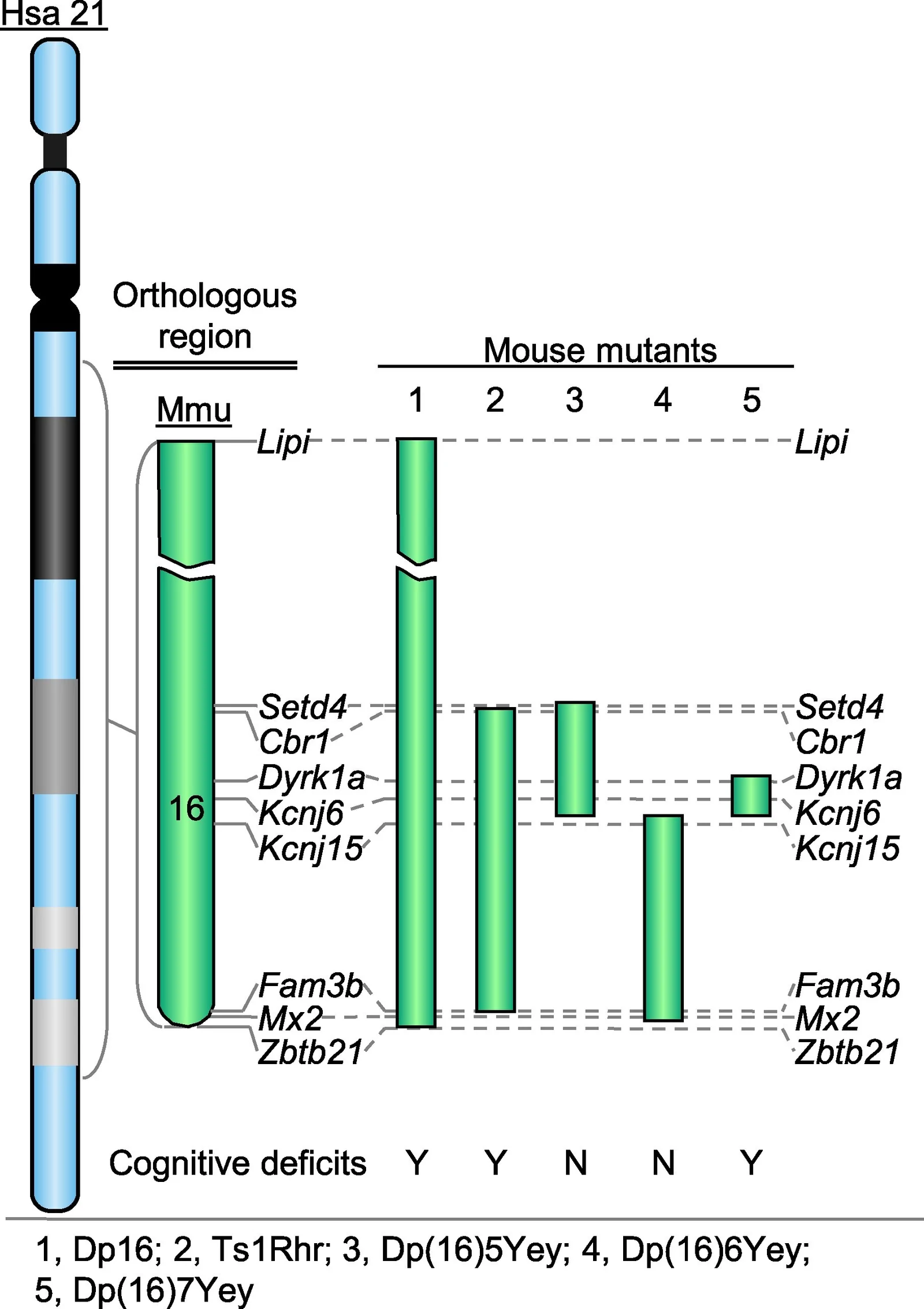
Mouse mutants and their cognitive phenotypes. The schematic illustrates the genomic positions, extents, and overlap of the duplicated intervals in Dp(16)5Yey, Dp(16)6Yey, Dp(16)7Yey, and Ts1Rhr mice. Hsa21 and the orthologous region on Mmu16 are shown. Dashed lines indicate the duplication endpoint genes. For comparison with additional duplication and deletion mouse models spanning the Hsa21-syntenic region on Mmu16, see previously described models (Xing et al. 2016; Herault et al. 2017; Folz et al. 2025). 1, Dp16; 2, Ts1Rhr; 3, Dp(16)5Yey; 4, Dp(16)6Yey; and 5, Dp(16)7Yey. Cognitive deficits: Y, yes; N, no.

The mouse DSCR spans the genomic interval between *Setd4* and *Mx2* on mouse chromosome 16 (Mmu16) (Fig. 1) (Olson et al. 2004; Jiang et al. 2015). An increased copy number of this genomic region has been shown to cause cognitive deficits in mice (Belichenko et al. 2009). To perform a systematic genetic dissection of this region using defined triplications, we subdivided the DSCR into 2 complementary segments: the *Setd4–Kcn*j6 interval and the *Kcnj15–Mx2* interval.

**Fig. 2.**
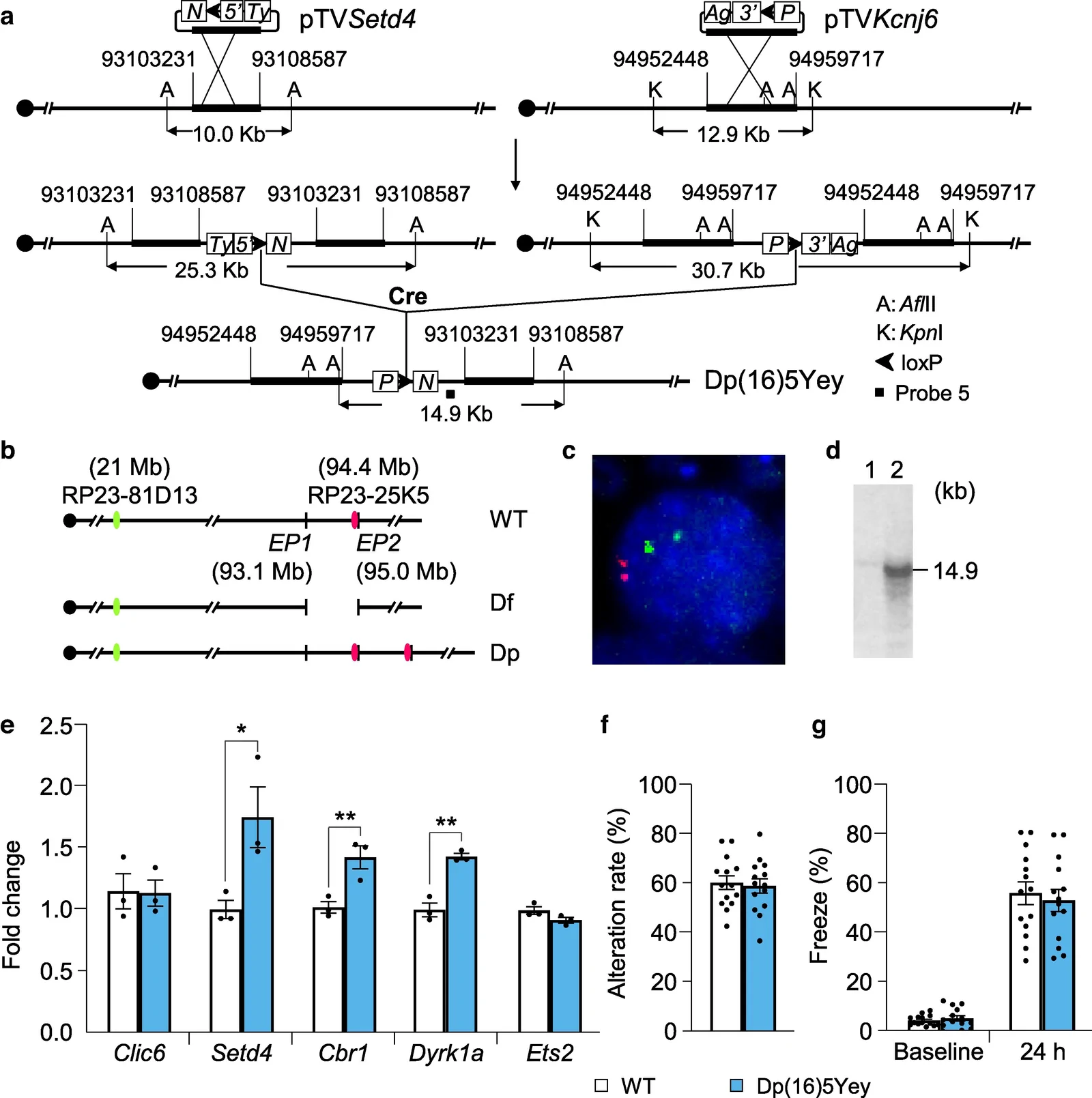
Generation and analysis of Dp(16)5Yey mice. a) Strategy to generate Dp(16)5Yey*. 5*′, 5′ half of a *HPRT* minigene; *3*′, 3′ half of a *HPRT* minigene; *N*, neomycin resistance gene; *P*, puromycin resistance gene; *Ty*, tyrosinase transgene; *Ag*, agouti transgene; arrowhead, *loxP*; A, *Afl*II; K, *Kpn*I. b) Genomic locations of BAC probes for FISH analysis. EP1, endpoint 1; EP2, endpoint 2. c) FISH analysis of interphase chromosomes prepared from Dp(16)5Yey/Df(16)5Yey ES cells. d) Southern blot analysis, using probe 5, of *Afl*II-digested tail DNA from a WT (lane 1) and a Dp(16)5Yey (lane 2) mouse. e) Gene expression fold changes of *Clic6*, *Setd4*, *Cbr1*, *Dyrk1a*, and *Ets2* in the cerebral cortex of Dp(16)5Yey mice (*n* = 3) over their WT littermates (*n* = 3). *Gapdh* was used as an internal control and is disomic in both Dp(16)5Yey and WT. f) Dp(16)5Yey (*n* = 14) and WT (*n* = 15) mice were examined in the T-maze. g) Dp(16)5Yey (*n* = 15) and WT (*n* = 14) mice were examined in a contextual fear-conditioning test. The percentages of time frozen before the foot shock and during the 24-h contextual tests are shown. *, *P* < 0.05; **, *P* < 0.01.

We first engineered Dp(16)5Yey mice, which carry a triplication spanning the *Setd4–Kcnj6* interval and encompass 15 Hsa21 gene orthologs (Fig. 1; Supplementary Table 1). To generate Dp(16)5Yey in mouse embryonic stem (ES) cells, MICER clones MHPN267e05 (pTV*Setd4*) and MHPP54c08 (pTV*Kcnj6*) were used to introduce loxP sites proximal to *Setd4* and distal to *Kcnj6*, respectively. Double-targeted ES cells were subsequently transfected with a Cre-expression vector to induce site-specific recombination, and clones carrying the correctly engineered duplication were identified by Southern blot analysis. The duplication was further confirmed by FISH (Fig. 2a–c). Mice carrying the engineered duplication were derived from these ES cells, verified by Southern blot analysis (Fig. 2d), and are hereafter referred to as Dp(16)5Yey mice. Dp(16)5Yey mice appeared overtly normal and exhibited Mendelian transmission ratios.

To determine whether copy-number variation resulted in corresponding changes in gene expression, we analyzed *Clic6, Setd4, Cbr1, Dyrk1a*, and *Ets2* mRNA levels in the cerebral cortex of Dp(16)5Yey using real-time quantitative PCR (RT-qPCR) (Fig. 2e). These genes were selected based on their genomic positions relative to the duplication breakpoints and the detectability of their expression in the cerebral cortex (Supplementary Table 1). The results demonstrated that transcript levels scaled with gene copy number, confirming dosage-dependent expression of these genes in the mutant mice.

To analyze the effects of increased dosage of the *Setd4–Kcnj6* interval on DS-associated cognitive phenotypes, we compared Dp(16)5Yey mice with wild-type (WT) littermate controls. No significant differences were detected between genotypes in either the T-maze or contextual fear-conditioning tests (*P* > 0.05; Fig. 2f and g). These results indicate that triplication of the *Setd4–Kcnj6* interval alone is not sufficient to elicit the cognitive phenotypes examined.

### Engineering and phenotypic analysis of mice carrying the triplication spanning the *Kcnj15–Mx2* genomic region

**Fig. 3.**
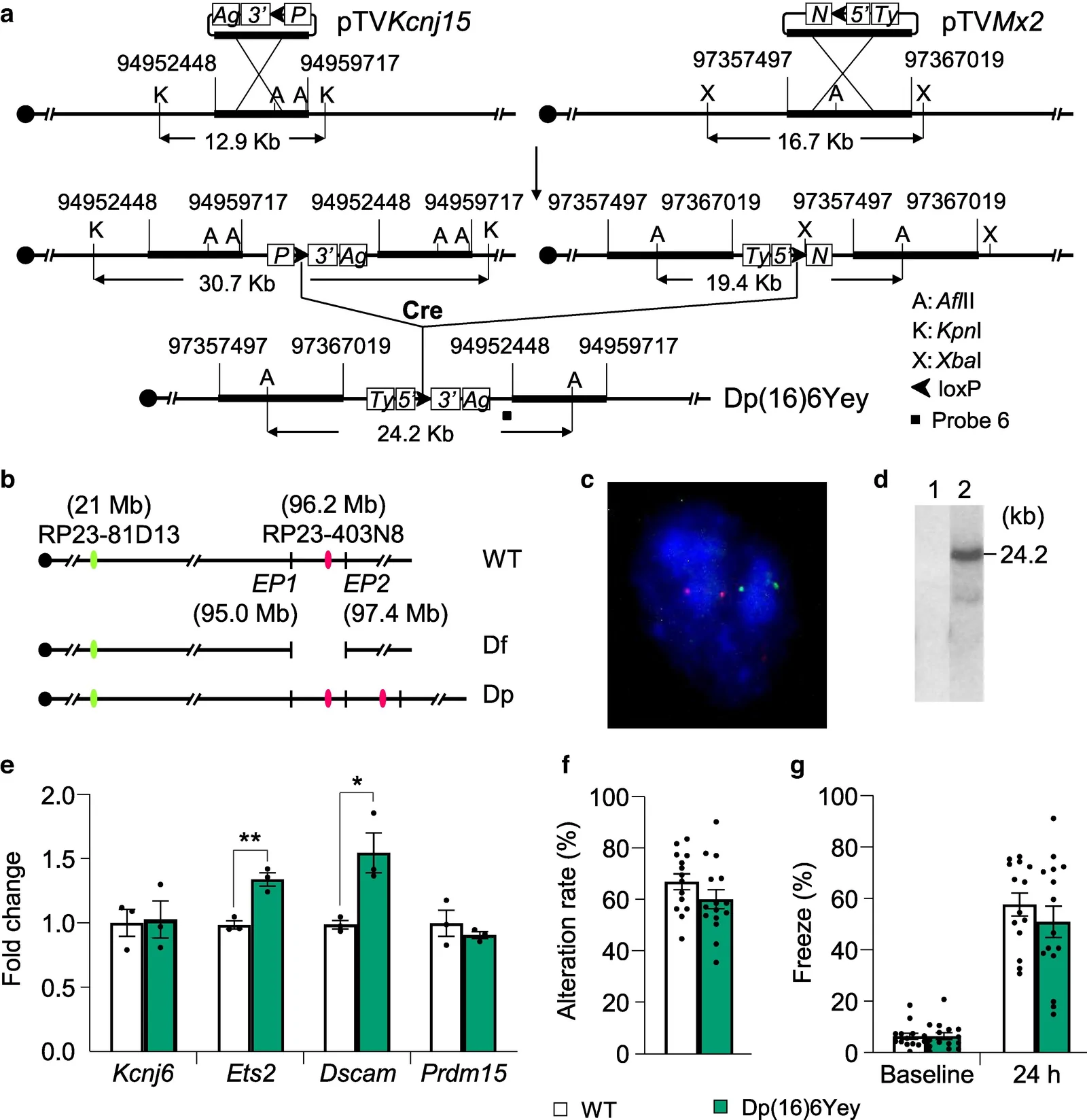
Generation and analysis of Dp(16)6Yey mice. a) Strategy to generate Dp(16)6Yey. For the definitions of the abbreviations, see the legend in Fig. 2a. A, *Afl*II; K, *Kpn*I; and X, *Xba*I. b) Genomic locations of BAC probes for FISH analysis. EP1, endpoint 1; EP2, endpoint 2. c) FISH analysis of interphase chromosomes prepared from Dp(16)6Yey/Df(16)6Yey ES cells. d) Southern blot analysis, using probe 6, of *Afl*II*-*digested tail DNA from a WT (lane 1) and a Dp(16)6Yey (lane 2) mouse. e) Gene expression fold changes of *Kcnj6*, *Ets2*, *Dscam*, and *Prdm15* in the cerebral cortex of Dp(16)6Yey mice (*n* = 3) over their WT littermates (*n* = 3). *Gapdh* was used as an internal control and is disomic in both Dp(16)5Yey and WT. f) Dp(16)6Yey (*n* = 16) and WT (*n* = 14) mice were examined in the T-maze. g) Dp(16)6Yey (*n* = 15) and WT (*n* = 14) mice were examined in a contextual fear-conditioning test. The percentages of time frozen before the foot shock and during the 24-h contextual tests are shown. *, *P* < 0.05; **, *P* < 0.01.

To further dissect the DSCR, we next engineered Dp(16)6Yey mice, which carry a triplication spanning the *Kcnj15–Mx2* interval and encompass 18 Hsa21 gene orthologs (Fig. 1; Supplementary Table 1). To generate Dp(16)6Yey in mouse ES cells, MICER clones MHPP54c08 (pTV*Kcnj15*) and MHPN178b08 (pTV*Mx2*) were used to introduce loxP sites proximal to *Kcnj15* and distal to *Mx2*, respectively. Following Cre-mediated recombination, correctly duplicated ES cell clones were identified by Southern blot analysis, and the duplication was independently confirmed by FISH (Fig. 3a–c). Mutant mice established using these ES cells were verified by Southern blot analysis (Fig. 3d) and are hereafter referred to as Dp(16)6Yey mice. Similar to Dp(16)5Yey mice, Dp(16)6Yey mice appeared overtly normal and exhibited Mendelian transmission ratios.

To determine whether copy-number variation resulted in corresponding changes in gene expression, we analyzed *Kcnj6*, *Ets2*, *Dscam*, and *Prdm15* mRNA levels in the cerebral cortex of Dp(16)6Yey using RT-qPCR (Fig. 3e). These genes were selected based on their genomic positions relative to the duplication breakpoints and the detectability of their expression in the cerebral cortex (Supplementary Table 1). The results demonstrated that transcript levels scaled with gene copy number, confirming dosage-dependent expression of these genes in the mutant mice.

To analyze the effects of increased dosage of the *Kcnj15–Mx2* interval on DS-associated cognitive phenotypes, we compared Dp(16)6Yey mice with WT littermate controls using the same behavioral assays employed for Dp(16)5Yey mice. No significant differences were observed between genotypes in the T-maze or contextual fear-conditioning tests (*P* > 0.05; Fig. 3f and g). These findings indicate that triplication of the *Kcnj15–Mx2* interval alone does not produce detectable cognitive deficits in the assays examined.

The results of the aforementioned analyses of the *Setd4–Kcnj6* and *Kcnj15–Mx2* regions using duplication models mirror those obtained using Dp16/Df(16)5Yey (i.e. Dp16/Del(16*Setd4–Kcnj6*)12Yey) and Dp16/Df(16)6Yey (i.e. Dp16/Del(16*Kcnj15–Mx2*)13Yey) mice (Jiang et al. 2015), with an additional important implication.

Because Ts1Rhr mice, carrying triplication of the DSCR interval between *Cbr1* and *Fam3b*, exhibit cognitive deficits in the T-maze spontaneous alternation test (Belichenko et al. 2009), our findings suggest that interactions between the *Setd4–Kcnj6* and *Kcnj15–Mx2* regions may contribute to the phenotypes observed in Ts1Rhr mice.

Notably, the Ts1Rhr model does not include triplication of *Setd4* or *Mx2*, whereas these genes are duplicated in Dp(16)5Yey and Dp(16)6Yey, respectively. Although the current work was not designed to directly assess the individual contributions of *Setd4* or *Mx2*, our findings suggest that dosage-sensitive loci within the *Setd4–Kcnj6* interval can modify the phenotypic effects associated with *Dyrk1a* and *Kcnj6* dosage increase. Thus, genes within this broader interval, including *Setd4* and/or other dosage-sensitive loci, may contribute to the complex genetic interactions underlying cognitive phenotypes (see Fig. 5).

### Engineering and phenotypic analysis of mice carrying a triplication of *Dyrk1a* and *Kcnj6*

Within the DSCR, *Dyrk1a* and *Kcnj6* are major candidate genes implicated in DS-associated cognitive deficits (de la Torre et al. 2014; Kleschevnikov et al. 2017) and have been proposed as therapeutic targets (Guedj et al. 2009; Cooper et al. 2012; de la Torre et al. 2014, 2016; Kleschevnikov et al. 2017; Souchet et al. 2019). These 2 genes are located immediately adjacent to each other within the DSCR (Fig. 1; Supplementary Table 1). *Dyrk1a* encodes a dual-specificity tyrosine-phosphorylation-regulated kinase that plays a critical role in multiple signaling pathways (White et al. 2003; Krumm and Duan 2019; Kempfer and Pombo 2020). *Kcnj6* encodes a G-protein-activated potassium channel that regulates neuronal excitability by mediating inhibitory signaling downstream of G-protein-coupled receptors for neuromodulators and neurotransmitters (Maass et al. 2019; Muller et al. 2019).

An interesting finding from our previous deletion-based (Df) analysis was that Dp16/Df(16)7Yey (i.e. Del(16*Dyrk1a–Kcnj6*)14Yey) mice exhibited no partial rescue of cognitive phenotypes, despite normalization of both *Dyrk1a* and *Kcnj6* copy number and without normalization of any other Hsa21 gene orthologs within the *Setd4–Mx2* interval. The result is important because it suggests therapeutic strategies to reduce the dosage effects of both genes at the same time may not be effective. This outcome was unexpected, because normalization of either *Dyrk1a* or *Kcnj6* alone had previously been shown to result in partial rescue of cognitive phenotypes (Jiang et al. 2015; Kleschevnikov et al. 2017).

**Fig. 4.**
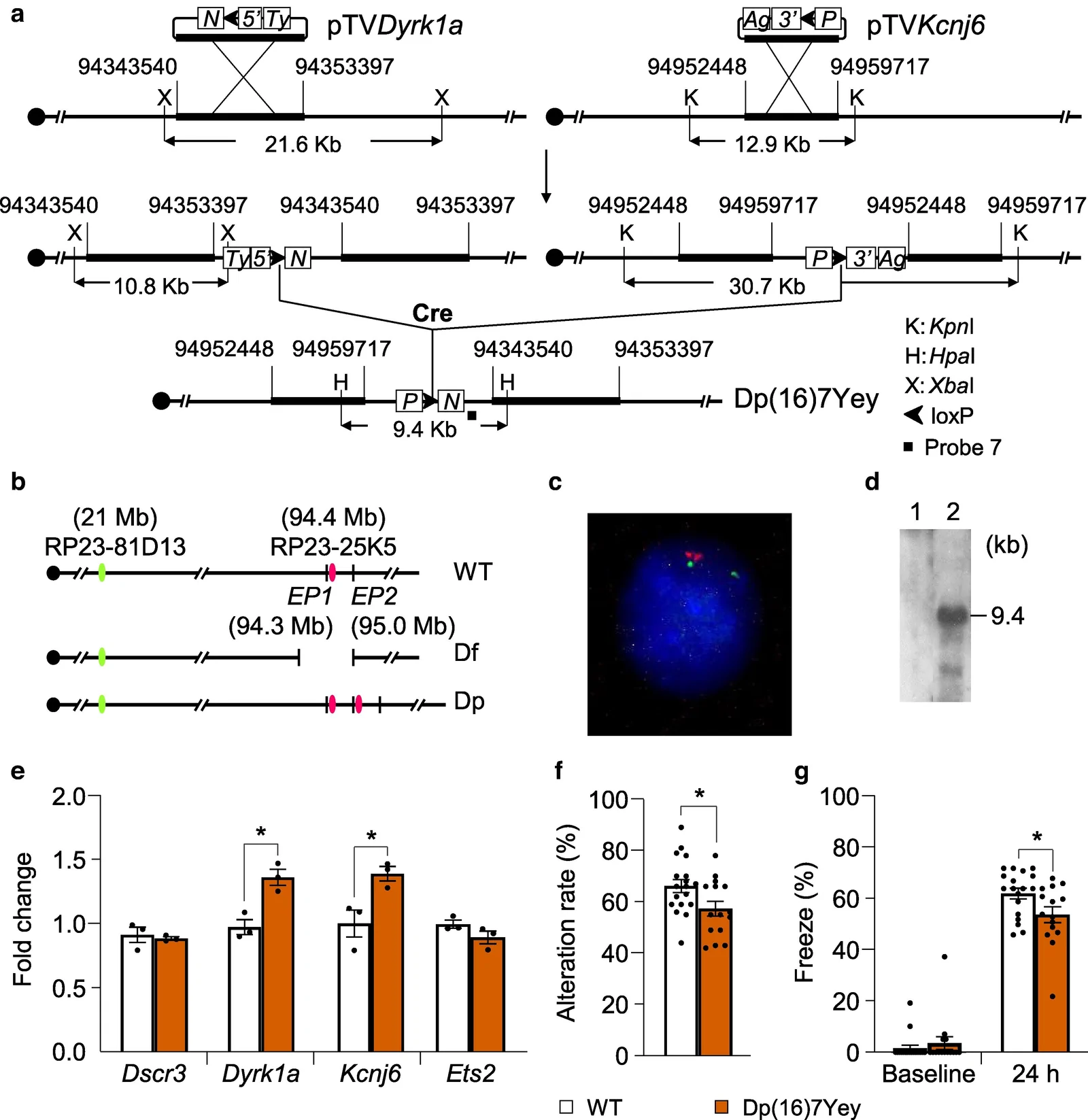
Generation and analysis of Dp(16)7Yey mice. a) Strategy to generate Dp(16)7Yey. For the definitions of the abbreviations, see the legend in Fig. 2a. K, *Kpn*I; H, *Hpa*I; and X, *Xba*I. b) Genomic locations of BAC probes for FISH analysis. EP1, endpoint 1; EP2, endpoint 2. c) FISH analysis of interphase chromosomes prepared from Dp(16)7/Df(16)7 ES cells. d) Southern blot analysis, using probe 7, of *Hpa*I*-*digested tail DNA from a WT (lane1) and a Dp(16)7Yey (lane 2) mouse. e) Gene expression fold changes of *Dscr3*, *Dyrk1a*, *Kcnj6*, and *Ets2* in the cerebral cortex of Dp(16)7Yey mice (*n* = 3) over their WT littermates (*n* = 3). *Gapdh* was used as an internal control and is disomic in both Dp(16)7Yey and WT. f) Dp(16)7Yey (*n* = 17) and WT (*n* = 11) mice were examined in the T-maze. g) Dp(16)7Yey (*n* = 15) and WT (*n* = 18) were examined in a contextual fear-conditioning test. The percentages of time frozen before the foot shock and during the 24-h contextual tests are shown. *, *P* < 0.05.

To further define the functional contribution of the *Dyrk1a*–*Kcnj6* interval within the DSCR, we engineered and analyzed mutant mice carrying a defined duplication of the *Dyrk1a–Kcnj6* interval. To generate Dp(16)7Yey mice, MICER clones MHPN85f19 (pTV*Dyrk1a*) and MHPP54c08 (pTV*Kcnj6*) were used to introduce loxP sites proximal to *Dyrk1a* and distal to *Kcnj6*, respectively, in mouse ES cells. Double-targeted ES cells were subsequently transfected with a Cre-expression vector to induce site-specific recombination, and clones carrying the correctly engineered duplication were identified by Southern blot analysis. The duplication was independently confirmed by FISH (Fig. 4a–c). Mice derived from these ES cells were verified by Southern blot analysis (Fig. 4d) and are hereafter referred to as Dp(16)7Yey mice. These mice appeared overtly normal and exhibited Mendelian transmission ratios.

To determine whether copy-number variation resulted in corresponding changes in gene expression, we analyzed *Dscr3*, *Dyrk1a*, *Kcnj6*, and *Ets2* mRNA levels in the cerebral cortex of Dp(16)7Yey using RT-qPCR (Fig. 4e). These genes were selected based on their genomic positions relative to the duplication breakpoints and the detectability of their expression in the cerebral cortex (Supplementary Table 1). The results demonstrated that transcript levels scaled with gene copy number, confirming dosage-dependent expression of these genes in the mutant mice.

To analyze the impact of increased dosage of the *Dyrk1a–Kcnj6* interval on DS-associated cognitive phenotypes, we compared Dp(16)7Yey mice with WT littermate controls. Dp(16)7Yey mice performed significantly worse than WT controls in both the T-maze and contextual fear-conditioning tests (*P* < 0.05; Fig. 4f and g), demonstrating that triplication of the *Dyrk1a–Kcnj6* interval is sufficient to elicit cognitively relevant phenotypes.

**Fig. 5.**
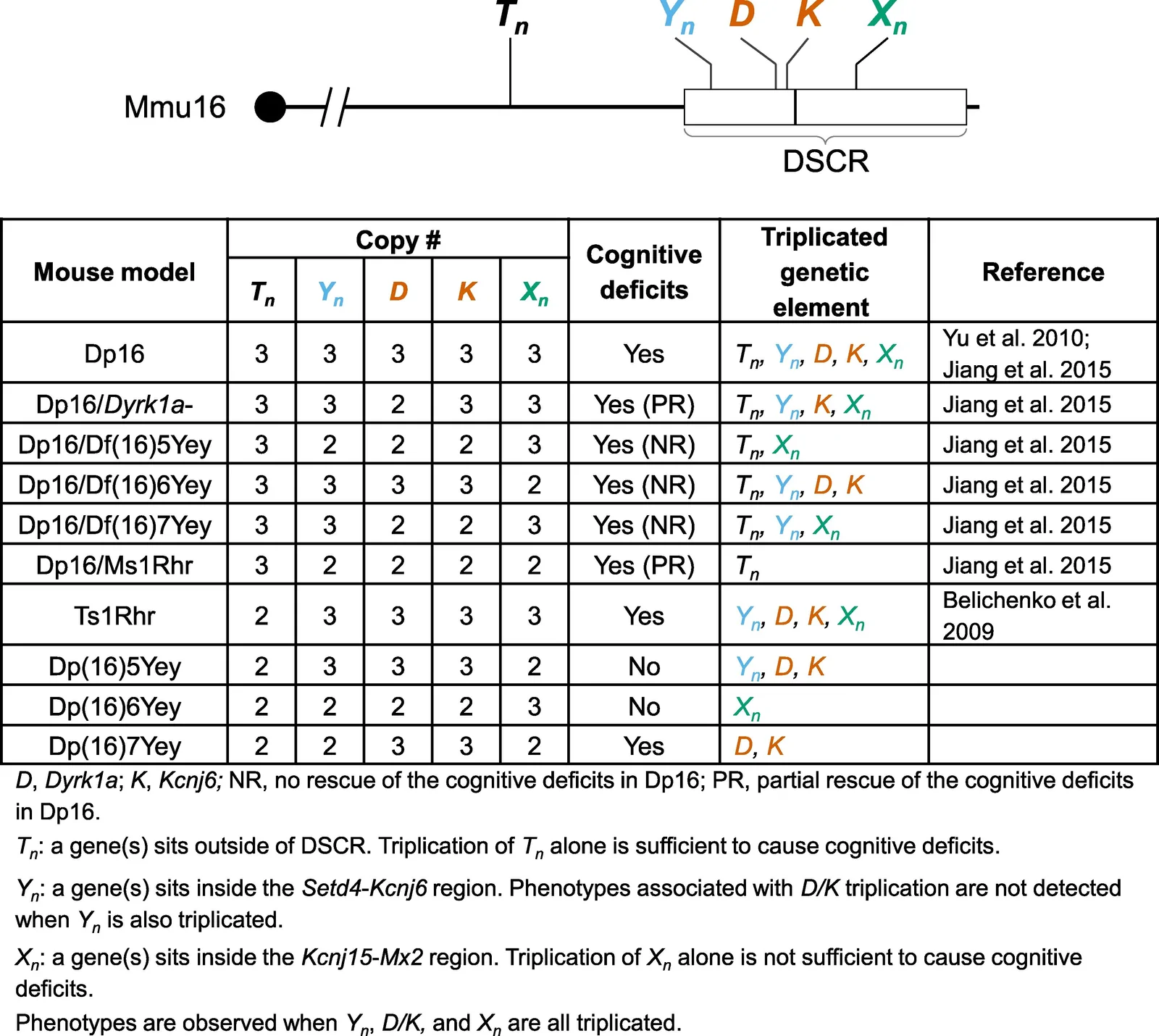
Triplicated genetic elements within the DSCR and beyond and associated phenotypic consequences in mouse models of DS.

Although simultaneous triplication of *Dyrk1a* and *Kcnj6* is sufficient to cause cognitive deficits in Dp(16)7Yey mice, this phenotype is absent in the larger Dp(16)5Yey duplication despite inclusion of the same core interval. This observation suggests that triplicated gene(s) outside the *Dyrk1a*–*Kcnj6* region (*Yn* in Fig. 5) can modify or mask the phenotypic effects associated with increased *Dyrk1a* and *Kcnj6* dosage. In contrast, cognitive impairment is observed in mice carrying the Ts1Rhr duplication (Belichenko et al. 2009), which encompasses both Dp(16)5Yey and Dp(16)6Yey, indicating that inclusion of the Dp(16)6Yey interval (*Xn* in Fig. 5) introduces additional dosage-sensitive interactions that overcome the masking effects observed in Dp(16)5Yey.

Consistent with this interpretation, prior studies of DSCR-targeted models showed that cognitive deficits were only partially rescued in Dp16/Ms1Rhr mice (Jiang et al. 2015), supporting a contribution from additional dosage-sensitive loci outside the canonical DSCR (*Tn* in Fig. 5) to DS-associated cognitive dysfunction. Furthermore, analysis of Dp16/Df(16)5Yey mice (Jiang et al. 2015) indicates that *Tn*, together with triplication of *Xn*, is associated with increased severity of cognitive phenotypes (Fig. 5). Similarly, phenotypic comparison between Dp16/Df(16)6 mice (Jiang et al. 2015), which retain triplication of *Tn,* and Dp(16)5Yey, suggests that *Tn* can reverse the masking effects of *Yn* on DS cognitive phenotypes from *Dyrk1a* and *Kcnj6* dosage increase (Fig. 5).

Our findings support the emerging view that DS phenotypes arise from complex interactions among multiple dosage-sensitive loci rather than from a single critical region alone. Previous studies have demonstrated multigenic contributions to diverse DS-associated phenotypes, including cognitive deficits (Duchon et al. 2021), congenital heart defects (Lana-Elola et al. 2024), craniofacial dysmorphology (Redhead et al. 2023), and skeletal abnormalities (Sloan et al. 2023). In this context, our study extends these observations by revealing a complex functional genetic architecture associated with the DSCR and by demonstrating that distinct duplication intervals can produce different cognitive phenotypes despite substantial overlap in gene content. The 5-locus model emerging from our genetic analyses further illustrates how interactions among dosage-sensitive loci can shape phenotypic outcomes. These findings support a model in which phenotypic expression depends not only on the presence of dosage-sensitive genes but also on their combinatorial interactions and broader genomic context. More broadly, our results underscore the importance of considering interacting dosage-sensitive loci when investigating the genetic basis of DS phenotypes and may have implications for the development of therapeutic strategies targeting multiple pathways.

Taken together, integration of the present duplication-based data with our previously generated deletion-based data (Jiang et al. 2015) reveals a comprehensive functional genetic architecture of the DSCR, as summarized in Fig. 5. Overall, this updated framework highlights the complexity of dosage-dependent interactions within and beyond the DSCR and has important implications for understanding its functional organization and for guiding the development of therapeutic strategies for DS-associated cognitive deficits.

## Supplementary Material

jkag173_Supplementary_Data

## Data Availability

Strains and plasmids are available upon request. The new mouse strains described in this study will be submitted to a public repository, such as the Jackson Laboratory, for deposition and broad distribution to the research community upon publication of the manuscript. The authors affirm that all data necessary for confirming the conclusions of the article are present within the article, figures, and table. Supplemental material is available at G3 online.
