# Supplementary material for: Duplication-based genetic dissection of the Down syndrome critical region reveals its complex functional organization
Source: G3 (Bethesda). 2026 Jul 1;16(9):jkag173. doi: 10.1093/g3journal/jkag173 (PMC13535499; doi:10.1093/g3journal/jkag173)
Supplement: Supplementary file 1 [file jkag173_supplementary_data.pdf]

Table S1. Orthologous genes in DSCR region on Mmu16 and Hsa21

	Names of mouse genes	Genome coordinates (Start position)	Names of human genes	Genome coordinates (Start position)	
	Setd4	93380345	*SETD4*	36034541	
	Cbr1	93404752	*CBR1*	36069941	
	Cbr3	93480107	*CBR3*	36135079	
	Dop1b	93508795	*DOP1B*	36156782	
	Morc3	93629009	*MORC3*	36320189	
	Chaf1b	93680801	*CHAF1B*	36385392	
	Cldn14	93715919	*CLDN14*	36460621	
	Sim2	93885963	*SIM2*	36699115	Dp(16)5Yey
	Hlcs	93929741	*HLCS*	36748625~~6~~	
	Ripply3	94129281	*RIPPLY3*	37006150	
	Pigp	94159622	*PIGP*	37062846	
	Ttc3	94171479	*TTC3*	37073226	
	Vps26c	94298583	*VPS26C*	37223420	
Dp(16)7Yey	*Dyrk1a*	94370869	*DYRK1A*	37365573	
	Kcnj6	94549495	*KCNJ6*	37607373	
	Kcnj15	95058417	*KCNJ15*	38155549	
	Erg	95160028	*ERG*	38367261	
	Ets2	95503274	*ETS2*	38805183	
	Psmg1	95781133	*PSMG1*	39174769	
	Brwd1	95793292	*BRWD1*	39184176	
	Hmgn1	95922788	*HMGN1*	39342315	
	Get1	95946607	*GET1*	39377698	
	Lca5l	95959605	*LCA5L*	39405728	
	Sh3bgr	96001670	*SH3BGR*	39445855	
	B3galt5	96037001	*B3GALT5*	39556442	Dp(16)6Yey
	Igsf5	96162868	*IGSF5*	39711771	
	Itgb2l	96223488	*ITGB2*	44885953	
	Pcp4	96268806	*PCP4*	39867438	
	Dscam	96392040	*DSCAM*	40010999	
	Bace2	97157942	*BACE2*	41167801	
	Mx1	97248235	*MX1*	41420020	
	Fam3b	97272165	*FAM3B*	41304212	
	Mx2	97337281	*MX2*	41361999	

All genomic coordinates reported in this study are based on the GRCm39 (mm39) mouse genome assembly.
